# Supplementary material for: Leprosy in an Adopted Woman Diagnosed by Molecular Tools: A Case Report from a Non-Endemic Area
Source: Pathogens. 2023 Jan 20;12(2):165. doi: 10.3390/pathogens12020165 (PMC9963898; doi:10.3390/pathogens12020165)

Supplementary Materials

Methods

Microscopical method

Microscopic examination of SSS, nasal swab, and skin biopsies was performed to reveal AFB. To perform the SSS, skin smears were taken bilaterally from the earlobes, elbows, and knees. A few microliters of dermal fluid, obtained from each site with aseptic precautions and a scalpel incision after skinfold compression, were transferred onto a microscope slide in a circular manner. Another slide was created for the nasal swab. The slides were dried at room temperature and fixed with 10% formalin (15 minutes). After rapid Kinyoun staining, the slides were examined with light microscopy using a 40X and 100X objective. Tissue biopsies were used for the histological diagnosis and to classify the disease. Histological examination used the skin biopsy specimens from multiple suspected sites, which were placed in 10% formalin and then stained with hematoxylin and eosin (H&E) and ZN (5%).

Serological method

Antibodies against phenolic glycolipid-I (PGL-I), the dominant glycolipid component of the M. leprae cell wall, were identified using the anti-PGL-I antibody ELISA test. The antigen semi-synthetic disaccharide (ND) of PGL-I coupled to BSA through a octyl (O) radical (ND-O-BSA) (kindley granted by dr. Spencer S.J.-Colorado University) was coated onto 96-well ELISA plates (ThermoFisher, Waltham, MA, USA) using 25 ng/well at 4°C overnight, as previously described [16]. After washing away the unbound antigen, the wells were blocked for one and a half hours at room temperature using 1% BSA (Sigma-Aldrich, Merck KGaA, Darmstadt, Germany) and 0.05% Tween 80 (Sigma-Aldrich, Merck KGaA, Darmstadt, Germany) in PBS (pH 7.4) (Sigma-Aldrich, Merck KGaA, Darmstadt, Germany). Then, three replicates of the serum samples at 1:300 dilution were added and incubated for two hours at room temperature. After washing with 0.05% Tween 80 in PBS (pH 7.4), 100µl/well at 1:10.000 dilution of the secondary anti-human polyvalent antibody (Sigma-Aldrich, Merck KGaA, Darmstadt, Germany) were added to each well and incubated for 30 minutes at 37°C. The enzyme reaction was developed with 50µl of the chromogenic alkaline phosphatase substrate (Sigma-Aldrich, Merck KGaA, Darmstadt, Germany) for 15 min in the dark. Absorbance at 405 nm was read using a ELx800 plate reader (Biotek, Winooski, VT, USA).

Table S1: List of primers used

Primer Forward (5'-3')	Primer Reverse (5'-3')	Targeted gene	Annealing temperature
GTCGAGGCGATCACGCCGC	CGACAATGAACCGATCAGAC	*rpoB*	**60°C**
ATGGTCTCAAACCGGTACATC	TACCCGGCGAACCGAAATTG	*gyrA*	**59°C**
GCAGGTTATTGGGGTTTTGA	CACCAGACACATCGTTGACG	*folP1*	**55°C**
GAGCCACTATTTCCCGACAA	CGTCGTCGATGAGCAAGTAA	*ml2535*	**58°C**

Figure S1: Sequences and chromatograms visualized on Codon Code aligner of the drug-resistant determining regions (indicated with the black double-arrow) of **A.** *rpoB,* **B.** *folP1* and **C.** *gyrA* amplified from a skin biopsy 3654-2136/21 and compared with the wild-type sequence and **D.** *ml2535* surrounding the specific mutation (c3016895a) found in the 1D genotype amplified from 3654-5546/21 and compared with the wild-type sequence. For genotyping, we used the primers previously described [18].

A. *rpoB*

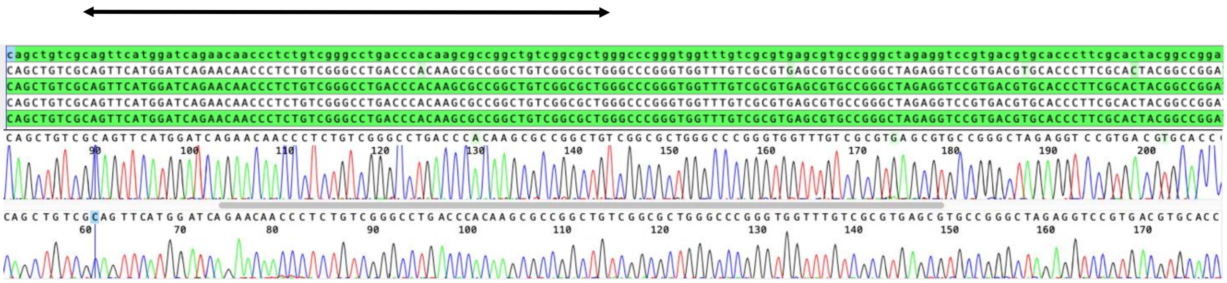

B. *folP1*

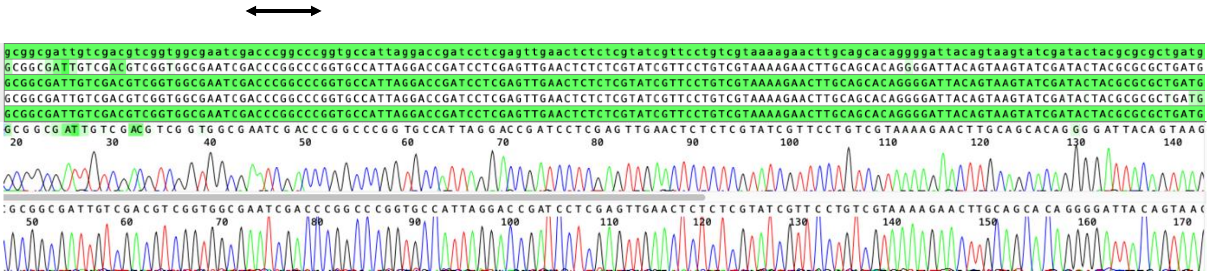

C. *gyrA*

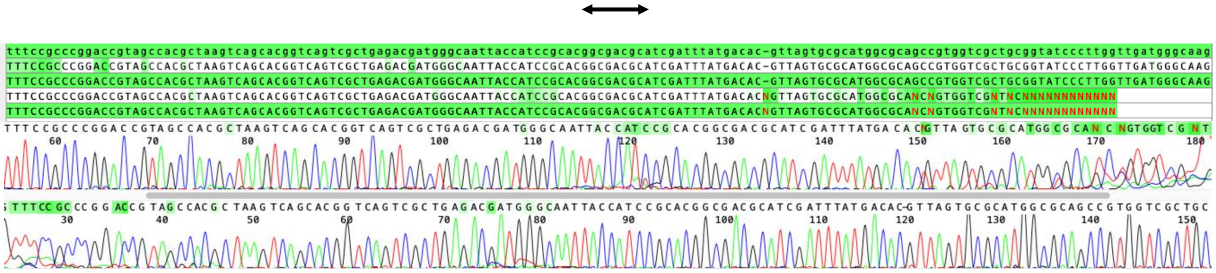

D. ml2535

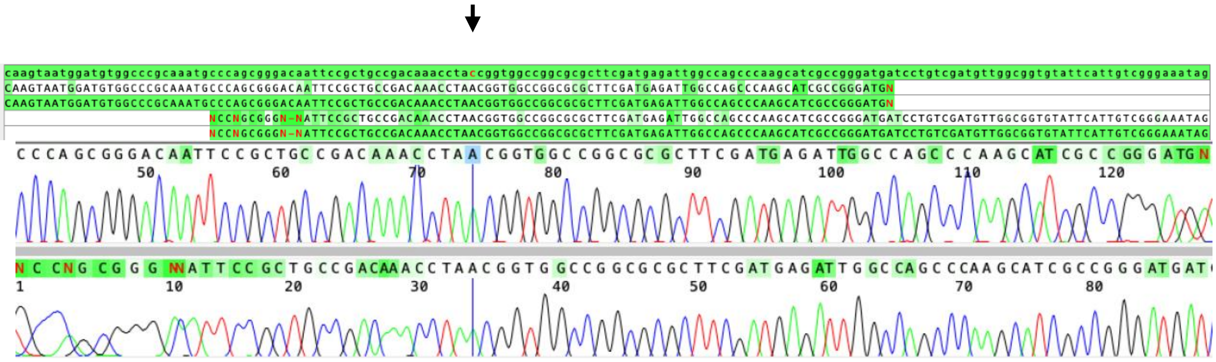

Supplement: Supplementary file 1 [file pathogens-12-00165-s001.zip › pathogens-2140174-supplementary.pdf]
